# Hydrolysis of Soybean Milk Protein by Papain: Antioxidant, Anti-Angiotensin, Antigenic and Digestibility Perspectives

**DOI:** 10.3390/bioengineering9090418

**Published:** 2022-08-26

**Authors:** Arijit Nath, Abubakar Saleh Ahmad, Abraham Amankwaa, Barbara Csehi, Zsuzsanna Mednyánszky, Emőke Szerdahelyi, Attila Tóth, Judit Tormási, Duy Hoàng Truong, László Abrankó, András Koris

**Affiliations:** 1 Department of Food Process Engineering, Institute of Food Science and Technology, Hungarian University of Agriculture and Life Sciences, Ménesi St 44, HU-1118 Budapest, Hungary; 2 Department of Refrigeration and Livestock Products Technology, Institute of Food Science and Technology, Hungarian University of Agriculture and Life Sciences, Ménesi út 43-45, HU-1118 Budapest, Hungary; 3 Department of Nutrition, Institute of Food Science and Technology, Hungarian University of Agriculture and Life Sciences, Somlói St 14-16, HU-1118 Budapest, Hungary; 4 Division of Clinical Physiology, Department of Cardiology, Faculty of Medicine, University of Debrecen, Móricz Zsigmond Str 22, HU-4032 Debrecen, Hungary; 5 Department of Food Chemistry and Analytical Chemistry, Institute of Food Science and Technology, Hungarian University of Agriculture and Life Sciences, Villányi út 35-43, HU-1118 Budapest, Hungary; 6 Institute of Biotechnology and Food Technology, Industrial University of Ho Chi Minh City, 12 Nguyen Van Bao, Ward 4, Go Vap District, Ho Chi Minh City 727000, Vietnam

**Keywords:** soybean milk protein, antioxidant capacity, anti-angiotensin activity, antigenic property, digestibility

## Abstract

The objective of the investigation was to understand the biochemical activities of hydrolysate of soybean milk protein (SMP). Hydrolysis was carried out by different concentrations of papain (0.008 g·L^−1^, 0.016 g·L^−1^, 0.032 g·L^−1^ and 0.064 g·L^−1^). The antioxidant capacity was measured by the ferric-reducing ability of plasma (FRAP) and 2,2-Diphenyl-1-picrylhydrazyl (DPPH) assays. The anti-angiotensin activity of hydrolysate was measured by the recombinant angiotensin converting enzyme and substrate Abz-FRK(Dnp)-P. The contributions of the Kunitz trypsin inhibitor (KTI) and Bowman–Birk inhibitor (BBI) on antigenicity, and the in vitro digestion of papain-hydrolyzed SMP were studied. Rabbit polyclonal anti-KTI and anti-BBI antibodies together with peroxidase-labelled goat anti-Rb IgG secondary antibody were used to identify the antigenicity of KTI and BBI in unhydrolyzed and papain-hydrolyzed SMP. The antioxidant capacity and anti-angiotensin activity of SMP were increased after the papain hydrolysis of SMP. The KTI- and BBI-specific antigenicity were reduced in SMP by increasing the concentration of papain. However, there was interaction between papain-hydrolyzed SMP and trypsin in native gel, while interaction with chymotrypsin was absent. The interaction between trypsin and SMP was reduced due to the hydrolysis of papain in a concentration-dependent manner. According to the in vitro gastrointestinal digestion simulation protocol (Infogest), the digestibility of SMP was not statistically increased.

## 1. Introduction

Over the past decade, several arguments have been raised about the consumption of dairy-based milk formulation because of unhealthy outcomes among infant to adult individuals [1]. As an alternative, cereal- or legume-based milk products came to the forefront. Presently, milk from leguminous soybean (*Glycine max*) has received great consideration [2]. Soybean milk protein (SMP) has been recognized as a high-quality source of protein because it contains all of the essential amino acids necessary for human diet and nutrition [3]. Contradictorily, soybean proteins are listed among the “big 8” allergens due to the presence of linear and conformational epitopes in protein structure. Different subunits of soybean proteins contain IgE- and IgG-binding B-cell epitopes [4]. Furthermore, the contribution of other characteristics and features of proteins, such as resistance to proteolysis by gastrointestinal proteases, may induce allergenic responses of proteins [5]. The presence of an intramolecular disulfide bond acts effectively to fold the protein and provide stability in the gastrointestinal tract. Stable proteins are likely to stay in the gastrointestinal tract for sufficient time and participate in an immunogenic reaction [6]. For example: soybean protein Kunitz trypsin inhibitor (KTI) consists of 181 amino acids and has 2 intrachain disulfide bridges. It has IgE-binding epitopes and primarily downregulates trypsin [7]. The Bowman–Birk inhibitor (BBI) is made up of 71 amino acids and has 7 intrachain disulfide bridges. BBI has two independent inhibitory binding sites, one for trypsin and another for chymotrypsin [7,8]. Soybean allergens can be classified to class 1 allergen and class 2 allergen based on sensitization route. Class 1 allergens mainly cause systemic symptoms, such as vomiting, diarrhea, atopy, urticaria and anaphylaxis. Class 2 allergens mainly cause oral allergy syndromes, however adverse cases, such as anaphylaxis with facial swelling, airway narrowing and breathing difficulties have been reported [9,10]. 

From the above discussion, it may seem that the reduction in the allergenic activity of soybean protein is a considerably challenging issue [11]. Frequently, reduction in allergenic activity in soybean proteins has been performed by acid treatment [12,13], ultra-heat treatment [14,15], extrusion [16], autoclaving [17,18], high hydrostatic pressure treatment [19,20], high-pressure homogenizer [21], controlled instantaneous pressure drop [22], micro-wave treatment [21,23], cold plasma treatment [24], pulsed ultraviolet treatment [24,25], γ-irradiation [24,26], ultrasonic treatment [21,27], microbial fermentation [28,29] and enzymatic hydrolysis [30,31]. Due to formation of toxic compounds, such as 1,3-dichloro-2-propanol and 3-chloro-1,2-propane-diol, acid treatment is not considerable in food products [32,33]. Several disadvantages of physical methods were also published [34]. It was reported that the binding capacity of IgE-specific antibodies and allergenic potency of the 2S-globulin of soybean protein were enhanced after heating at a temperature of 80 °C [35]. Similarly, the IgE-binding ability of Gly m Bd 30K protein (a major soybean allergen) was increased due to combined high-pressure and heat treatment [36]. In another investigation, it was found that heat treatment did not affect the binding capacity of glycinin with IgE [37]. Furthermore, it was reported that heat treatment is a considerable energy-intensive and costly process, and has a negative influence on the nutrient quality of soybean-based products [38]. Among all possibilities, biological routes, i.e., microbial fermentation and enzymatic hydrolysis of SMP, have been considered as “safe”. Hydrolysis of protein can be controlled by the selection of a specific enzyme, reaction condition and operating parameters [39,40]. Furthermore, several functional properties can be improved by the enzymatic hydrolysis of protein [41,42]. Hydrolysis of SMP by enzyme produces peptides with unique biological activity. 

Food protein-derived bioactive peptides with unique biological values have been distributed to large numbers of consumers [43,44]. Soybean protein-derived peptides play an important role in the regulation of metabolic pathways and have a significant contribution in the modulation of metabolic syndrome [45]. A cluster of biochemical, physiological and clinical abnormalities is associated with metabolic syndrome that leads to several health hazards and may even cause death [46]. The circulatory hormone angiotensin II, an important effector of the renin–angiotensin system has an influence on oxidative stress and inflammation [47] through the activation of pro-inflammatory genes (nuclear factor-κB regulated genes) and the formation of pro-inflammatory elements (tumor necrosis factor-α, interleukin-1β, interleukin-6, interleukin-8, monocyte chemoattractant protein-1 and transforming growth factor-β) [48,49]. From the above discussion, it may be realized that the modulation of the activity of the renin–angiotensin–aldosterone system can decrease the risks and complications of metabolic abnormalities. Presently, a great attention has been placed for developing food protein-derived bioactive peptides with anti-angiotensin II activity and antioxidant capacity as an alternative to renin blocker, angiotensin-converting enzyme inhibitor and angiotensin II receptor blocker [50].

Several studies have been performed on the hydrolysis of soybean protein and the development of bioactive peptides from soybean meal [40,51,52], soybean protein concentrate [39,53], soybean protein isolate (SPI) [53,54], soybean flour [55,56] and soybean protein hydrolysate [57,58] through different serine proteases, which generally creates peptides with bitter taste [59,60]. Applications of cysteine proteases, such as papain (EC 3.4.22.2) from papaya latex, bromelain from pineapple stem (EC 3.4.22.32) and fruit (EC 3.4.22.33), ficin (EC 3.4.22.3) from the latex of fig, zingibain (EC 3.4.22.67) from ginger, actinidin (EC 3.4.22.14) from kiwifruit and aspartic protease cardosin (E.C.3.4.23) from tobacco in the food industry are acceptable because they do not create undesirable organoleptic properties [61,62]. Previously, some researchers used papain to produce peptides with functional activities from SMP [63] and SPI [64,65,66,67,68,69,70]. 

In the present investigation, SMP was hydrolyzed with different concentrations of papain in a batch-mode process. Several biological aspects, such as antioxidant capacity, anti-angiotensin activity and antigenicity for KTI and BBI of enzyme-treated soybean milk were evaluated. In a later exercise, the digestibility of protein in papain-treated soybean milk was analyzed by in vitro gastrointestinal digestion simulation protocol (Infogest). 

## 2. Materials and Methods

### 2.1. Soybean Milk 

Ultra-heat-treated (UHT) soybean milk was purchased from a local supermarket in Budapest, Hungary. The concentration of protein, fat and carbohydrate in soybean milk were measured before papain treatment. 

### 2.2. Chemicals and Reagents

Bradford reagent, BBI (T9777), KTI (T2327), trypsin (T-4665), α-amylase (A3176), pepsin (P1428), pancreatin (P7545), porcine bile extract (B8631), o-phthalaldehyde (OPA), 3,3′-Dithiodipropionic acid, bovine serum albumin, 2,4,6-Tris(2-pyridyl)-s-triazine, 2,2-diphenyl-1-picrylhydrazyl, N-acetyl-DL-phenylalanine β-naphthyl ester, N,N-dimethylformamide, tetrazotized o′-dianisidine-zinc chloride, 4-chloronaphtol, hydrogen peroxide, methanol, ethanol, fast blue B salt, hammersten-type casein, cysteine and phosphate buffered saline solution were purchased from Sigma–Aldrich (Sigma–Aldrich, Schnelldorf, Germany). Abz-FRK(Dnp)-P was purchased from Peptide 2.0 Inc., Virginia, VA, USA. Bovine α-chymotrypsin (27270) was purchased from Fluka, Germany. Acetic acid, ascorbic acid, di-sodium hydrogen phosphate, ferric chloride, hydrochloric acid, sodium acetate, sodium chloride, potassium-dihydrogen phosphate, potassium chloride, sodium di-hydrogen phosphate, sodium hydroxide, trichlorocacetic acid (TCA), potassium sulfate, copper sulfate, sulfuric acid, phenolphthalein, tween-20, citric acid, phenol, glucose, amyl-alcohol, ethylenediaminetetraacetic acid and zinc chloride were procured from Merck (Merck, Darmstadt, Germany). Sodium-dodecyl sulphate, 4-chloro-1-naphthol, acrylamide, ammonium persulfate, bis-acrylamide, tetramethylethylenediamine, tris-(hydroxymethyl)-aminomethane hydrochloride (TRIS HCl), glycine, glycerol, isopropanol, 2-mercaptoethanol, coomassie blue stain R250 and bromophenol blue were purchased from Bio-Rad (Bio-Rad, Hercules, CA, USA). Lyophilized papain (RM6518) was purchased from Himedia, India. All chemicals used in experiments were analytical grade. Recombinant angiotensin converting enzyme (ACE) was received from Division of Clinical Physiology, Institute of Cardiology, University of Debrecen, Hungary. Peroxidase-labelled goat anti-rabbit (Rb) Immunoglobulin G (IgG) secondary antibody was procured from Sigma–Aldrich (Sigma–Aldrich, Schnelldorf, Germany). Rabbit polyclonal antibodies against KTI and BBI were received from the Food Science Research Group, Institute of Food Science and Technology, Hungarian University of Agriculture and Life Sciences, Budapest, Hungary. Milli-Q ultrapure deionized water (18.2 MΩ·cm) was obtained from Milli-Q Synergy/Elix water purification system (Merck-Millipore, Molsheim, France) and used in all experiments. 

### 2.3. Hydrolysis of SMP by Papain

Papain hydrolysis of SMP was performed in a laboratory-scale bioreactor equipped with pH and temperature sensors (Solida Biotech, München, Germany). Prior to enzymatic reaction, 0.09 g of lyophilized papain (~30,000 USP units·mg^−1^) was dissolved in 10 mL of sterile de-ionized water, pH 7 in room temperature (RT) (~25 °C) and aseptic condition. Subsequently, papain solution was filtered through 0.22 μm of poly-ether sulfone syringe filter (VWR International, Radnor, PA, USA) in aseptic condition. In the bioreactor, 500 mL of soybean milk, pH 7 was pre-incubated to reach the reaction temperature 50 °C. Individual batch-mode protein hydrolysis was performed in the bioreactor with different concentrations of papain. For that purpose, 450 µL, 900 µL, 1.8 mL and 3.6 mL of papain solution from stock papain solution were aseptically injected to 500 mL of soybean milk in the bioreactor. Therefore, initial concentrations of papain in the hydrolysis reaction were 0.008 g·L^−1^, 0.016 g·L^−1^, 0.032 g·L^−1^ and 0.064 g·L^−1^. They are designated as SMP-0.008, SMP-0.016, SMP-0.032, and SMP-0.064, respectively. Based on our previous knowledge, we did not use concentration of papain greater than 0.064 g·L^−1^ for hydrolysis of SMP, to avoid protein coagulation [63]. Hydrolysis of SMP by papain was performed at a temperature of 50 °C for 10 min by batch-mode process. During protein hydrolysis reaction, pH of soybean milk in the bioreactor was maintained constant 7.0 by addition of 2.0 N of sodium hydroxide or hydrochloric acid in a programmed way. After 10 min of protein hydrolysis reaction, 25 mL of reaction mixture was collected in a sample tube from the bioreactor by a syringe. Sample tubes were immediately placed in a water bath (Thermo Scientific^TM^, Waltham, MA, USA) at a temperature of 70 °C for 30 min to inhibit the activity of papain [63].

### 2.4. Analytical Methods

#### 2.4.1. Molecular Weight of SMP and Peptides by Sodium Dodecyl Sulfate-Polyacrylamide Gel Electrophoresis (SDS-PAGE)

To understand the molecular weight of SMPs and peptides from SMPs, SDS-PAGE was performed by a vertical electrophoresis system (Bio-Rad mini protein Tetra system, Bio-Rad, USA). Concentrations of running gel and stacking gel were 15% and 6%, respectively, in SDS-PAGE protocol. Electrophoretic separation of proteins and peptides was performed by constant 200 V. Dilution of samples was performed with laemmli sample buffer (2X) and 10% 2-mercaptoethanol. Appropriately diluted 10 µL of samples were loaded in individual well within stacking gel. Standard proteins (precision plus protein standards, Bio Rad, USA) with molecular weight range 250–10 kDa, KTI and BBI were used for comparison purpose. Gel running was performed at RT for 60 min. Gel staining was performed with 0.2% Coomassie Brillant Blue R250 in 9% (volume basis) acetic acid (96%) and 45% ethanol for 30 min at RT. De-staining of gel was performed with 50% (volume basis) methanol-water and 10% (volume basis) acetic acid at RT. An image of the gel was taken using a Gel Doc System 2000 (Bio-Rad, CA, USA) [71]. The image was imported into the freeware program ImageJ, version 1.5 (National Institutes of Health (NIH), Bethesda, MD, USA) for quantifying the band intensities of different proteins.

#### 2.4.2. Immunoblotting

Proteins from gels of SDS-PAGE were transferred onto a polyvinylidene difluoride (PVDF) membrane with pore size 0.45 µm (Merck-Millipore, France) by a trans blot semi-dry transfer cell (Bio-Rad, USA). According to our previous experience, it was operated with 0.25 V and 0.8 mA·cm^−2^ for 60 min at RT. Rb polyclonal anti-KTI and anti-BBI antibodies together with peroxidase-labelled goat anti-Rb IgG secondary antibody were used to identify the antigenicity of proteins in unhydrolyzed soybean milk and papain-treated soybean milk. After the blotting procedure, the pre-stained molecular weight standard was not visible on the gel, but it was clearly visible on the PVDF membrane. The binding patterns of the antibody were noted using a substrate solution, containing hydrogen peroxide, 4-chloronaphtol and ethanol in 16 mM phosphate buffered saline solution, pH 7.2. Image analysis of blot was carried out by Gel Doc 2000 system (Bio-Rad, Hercules, CA, USA) [72]. 

#### 2.4.3. Degree of Hydrolysis (DH) of SMP

DH of SMP was measured after inactivation of the activity of papain, the subsequent step of papain hydrolysis of SMP. Temperature of samples was reduced from 70 °C to RT. Thereafter, 5 mL of reaction mixture was mixed with 5 mL of 10% TCA and incubated for 30 min at RT [73]. Subsequently, the sample with TCA was centrifuged for 10 min by a laboratory centrifuge (Hermle, Gosheim, Germany). During centrifugation, rotor speed and temperature were 5000 rpm and 4 °C, respectively. In the next step, suspended solids were removed by Whatman filter paper with pore size 0.2 µm and supernatant liquid was considered to measure the DH. Concentration of TCA-soluble nitrogen in clarified liquid and total nitrogen in soybean milk were determined by the Kjeldahl method [74]. DH of SMP was determined by the amount of soluble nitrogen in 5% TCA with respect of total SMP [75]. The following equation was considered to calculate the DH of SMP.

$$\mathrm{DH}\left(\%\right)=\frac{\mathrm{TCA}\;\mathrm{soluble}\;N_{2}\;\mathrm{after}\;\mathrm{protein}\;\mathrm{hydrolysis}\;-\;\mathrm{TCA}\;\mathrm{soluble}\;N_{2}\;\mathrm{before}\;\mathrm{protein}\;\mathrm{hydrolysis}}{\mathrm{Total}\;N_{2}\;\mathrm{in}\;\mathrm{soybean}\;\mathrm{milk}\;-\;\mathrm{TCA}\;\mathrm{soluble}\;N_{2}\;\mathrm{before}\;\mathrm{protein}\;\mathrm{hydrolysis}}\times 100$$

#### 2.4.4. Determination of Protein Concentration

Concentration of total nitrogen as well as protein in unhydrolyzed soybean milk and supernatant after TCA precipitation were quantified by the Kjeldahl method [74]. A programmed Kjeldahl nitrogen analyzer along with a block digestion unit (C. Gerhardt GmbH & Co. KG, Königswinter, Germany) was used in this investigation. During conversion of the amount of nitrogen to concentration of protein, a conversion factor of 6.25 was considered.

Concentrations of protein of papain, unhydrolyzed soybean milk and after inactivation of papain in soybean milk were measured by the Bradford method [76]. One hundred µL of appropriately diluted samples were mixed with 3 mL of Bradford reagent and incubated at RT for 30 min in a water bath. Bovine serum albumin was considered as a standard. Wavelength 595 nm was used for colorimetric analysis. A UV-Vis spectrophotometer (Thermo Scientific^TM^, Waltham, MA, USA) was used for that purpose. 

#### 2.4.5. Determination of Total Carbohydrate Concentration

Concentration of total carbohydrate in unhydrolyzed soybean milk was quantified by the phenol sulfuric acid method [77]. Glucose was used as a reference in the experimental protocol. Colorimetric determination was performed with wavelength 490 nm. A UV-Vis spectrophotometer (Thermo Scientific^TM^, Waltham, MA, USA) was used. 

#### 2.4.6. Determination of Total Fat Concentration

Concentration of total fat in unhydrolyzed soybean milk was quantified by the Gerber method [78]. In a butyrometer, 10 mL of sulfuric acid was mixed with 10.75 mL of unhydrolyzed soybean milk. Subsequently, 1 mL of amyl alcohol was added to the mixture and the solution was shaken gently. The butyrometer was placed in a centrifuge (Hermle, Gosheim, Germany) and centrifugation was performed by 1100 rpm for 4 min. Concentration of fat in unhydrolyzed soybean milk was determined by the position of the fat layer in butyrometer.

#### 2.4.7. Determination of the Activity of Papain

Activity of papain prior to hydrolysis of SMP was measured considering Hammersten-type casein as substrate. Substrate solution was prepared using 1 g of casein in 50 mL of 0.05 M Dibasic sodium phosphate. During preparation of casein solution, 0.05 M citric acid was used and the final pH was 6.0 ± 0.01. An appropriately diluted papain solution in phosphate-cysteine disodium ethylenediaminetetraacetate buffer was added to 5 mL of 1% (*w*/*v*) casein substrate solution, pH 6.0 ± 0.01. Casein hydrolysis was performed at a temperature of 37 °C for 20 min. The reaction was stopped by the addition of 3.0 mL of 30% (*w*/*v*) TCA solution and incubated for 30 min at RT. Subsequently, the reaction mixture was centrifuged at a temperature of 5 °C for 10 min by a laboratory centrifuge (Hermle, Gosheim, Germany) and supernatant was filtered by 0.2 µm poly-ether sulfone (PES) membrane filter (Merck-Millipore, France). Concentration of tyrosine was measured with wavelength 280 nm. A UV-Vis spectrophotometer (Thermo Scientific^TM^, Waltham, MA, USA) was used for that purpose. One USP unit of papain activity represents the equivalent release of 1 mg of tyrosine from a casein substrate solution [79].

#### 2.4.8. Determination of the Antioxidant Capacity

After inactivation of papain, enzymatic reaction mixture and unhydrolyzed soybean milk were centrifuged in a laboratory centrifuge (Hermle, Gosheim, Germany). Centrifugation was performed with 5000 rpm at a temperature of 4 °C.

##### Ferric-Reducing Ability of Plasma (FRAP) Assay

The antioxidant capacity of supernatants was measured using the ferric-reducing ability of plasma (FRAP) assay, considering ascorbic acid as a standard. One hundred µL of sample was mixed with 2.9 mL of FRAP reagent (300 mM acetate buffer, pH 3.6: 20 mM ferric chloride: 10 mM 2,4,6-Tris(2-pyridyl)-s-triazine with 40 mM hydrochloric acid = 10:1:1 (volume basis)) and incubated at a temperature of ~35 °C for 30 min in a water bath (Thermo Scientific^TM^, Waltham, MA, USA). Colorimetric determination was performed in RT with a UV-Vis spectrophotometer (Thermo Scientific^TM^, Waltham, MA, USA). Spectrophotometric measurement was performed with wavelength 593 nm [80]. 

##### 2,2-Diphenyl-1-Picrylhydrazyl (DPPH) Radical-Scavenging Assay

DPPH Radical-Scavenging activity was measured in supernatants of all samples. A total of 8.8 mg of DPPH was dissolved in 20 mL of 98% ethanol, considered as DPPH stock solution. Thereafter, 7.5 mL of freshly prepared DPPH stock solution was mixed with 67.5 mL of ethanol to prepare 6 × 10^−5^ M DPPH-ethanol working reagent. The assay was performed with 100 µL of supernatant and 3.9 mL of DPPH-ethanol working reagent. Control sample was prepared with 100 µL of ethanol and 3.9 mL of 6 × 10^−5^ M DPPH-ethanol working reagent. Thereafter, samples were incubated at RT in a dark condition for 15 min. The absorbance was measured with 517 nm by a UV-Vis spectrophotometer (Thermo Scientific^TM^, Waltham, MA, USA). The percentage radical-scavenging activity of the sample was measured based on control solution (without sample) [81]. 

#### 2.4.9. Determination of ACE Inhibitory Activity

Anti-ACE activity of unhydrolyzed and papain-hydrolyzed SMP was measured by a quenched fluorescent substrate Abz-FRK(Dnp)-P (Peptide 2.0 Inc., Virginia, VA, USA) and recombinant ACE. An assay mixture (200 µL final volume) was contained 250 mM sodium chloride, 100 mM TRIS-HCl (pH 7.0), 10 µM zinc chloride, 15 µM Abz-FRK(Dnp)-P and recombinant ACE (about 100-fold dilution). Activities of ACE without SMP represented the uninhibited ACE, expressed as 100% of activity. For inhibitory activity assay, ACE was pre-incubated with papain-hydrolyzed soybean milk samples (protein concentration 0.01–1 mg·mL^−1^) for 10 min at RT to facilitate steady state binding of the potential inhibitor to ACE. Subsequently, 15 µM substrate was added to the assay mixture and ACE activity was measured. Fluorescent intensity measurement was performed by a fluorescent plate reader (BMG Labtech, Ortenberg, Germany) at a temperature of 37 °C in Corning 96 wells black and flat bottom plates (Corning, New York, NY, USA). Excitation and emission wavelengths were 340 nm and 405 nm, respectively, during measurement. Fluorescence was recorded at each minute for at least 20 cycles and data points were plotted as the function of time. These plots were fitted by a linear fit, and the slope was used to estimate enzyme activity. The level of inhibition was calculated based on the percentage of uninhibited activity in each plate compared to inhibited ACE activity (100% activity) [82]. ACE activity was plotted as the function of concentration of protein in unhydrolyzed and papain-hydrolyzed soybean milk samples. The value of IC_50_ of each sample was determined by the nonlinear fit. Furthermore, activity of ACE and corresponding substrate concentration were used to determine the values of *K_M_* and *V_max_* by the Lineweaver Burk Plot. 

#### 2.4.10. Inhibitory Activities of KTI and BBI in Native (Non-Denaturing Condition) Polyacrylamide Gels

Two native (non-denaturing conditions) polyacrylamide gel electrophoresis (PAGE) processes were performed for trypsin and chymotrypsin. Native gel running was performed by a vertical gel electrophoresis system (Bio-Rad mini protein Tetra system, Bio-Rad, USA). In this electrophoretic method, concentrations of stacking and running gels were 6% and 15%, respectively, without SDS in gels and running buffer. Samples (unhydrolyzed SMP and papain-hydrolyzed SMP) were dissolved in 1 mL of running buffer (3.03 g Tris, 14.4 g glycine in 1000 mL of distilled water) together with 200 mg of saccharose. Native polyacrylamide gel electrophoretic separation of proteins and peptides was performed by constant 200 V at RT for 60 min. Protein staining and de-staining were performed similarly to SDS-PAGE [83]. 

For inhibitory activity staining after the native gel running, gels were washed with DI water and incubated in 0.3 M phosphate buffer (pH 7.5) containing 0.3 g·L^−1^ of bovine trypsin or chymotrypsin at a temperature of ~42 °C for 30 min. Gel staining was performed by freshly prepared staining solution at RT for 10 min. Detailed protocol for gel staining solution is herein. A total of 25 mg of N-acetyl-DLphenylalanine β-naphthyl ester chromogenic substrate was dissolved in 10 mL of N,N-dimethylformamide, and 42 mg of o-Dianisidine bis(diazotized) zinc double salt (Fast blue B salt) was dissolved in 38.5 mL of distilled water, while 0.3 M phosphate buffer was used to adjust the volume up to 77 mL. These solutions were mixed immediately to prepare gel staining solution and working staining solution was poured on the gel. The stained gels were rinsed in distilled water at RT for 5 min for removal of unbounded stain and gels were stored in 1% acetic acid. Activity of trypsin or chymotrypsin in gel was visualized in a dark violet or pink background [84].

#### 2.4.11. In Vitro Digestion

In vitro digestion was performed to evaluate protein digestibility of soybean milk hydrolyzates using the Infogest consensus protocol [85]. For this purpose, a scaled down version of the original protocol was used for 1 g (1 mL) of sample size, containing ~40 mg of protein. Blank digestion was carried out using protein-free biscuit (concentration of protein 0.03% (*wt*/*wt*)). The composition of protein-free biscuit was mentioned elsewhere [86]. In blank digestion, 1 g of protein-free biscuit was used as a sample. Simulated salivary fluid (SSF), simulated gastric fluid (SGF), simulated intestinal fluid (SIF) were prepared beforehand. According to the Infogest guideline, pH test was performed to appropriately adjust the pH phases (oral phase 7, gastric phase: pH 3, intestinal phase: pH 7) during in vitro digestion simulation of unhydrolyzed SMP and papain-hydrolyzed SMP. According to the Infogest guideline, pH test was performed without gastrointestinal enzymes in simulated digestion fluids. For pH adjustment, 6 M hydrochloric acid and 1 M sodium hydroxide solutions were used. Enzyme activity was measured according to recommendations [87]. Steps of the Infogest protocol briefly: 1 g of sample was accurately measured into a pre-weighed 50 mL centrifuge tube. For oral phase, 0.7 mL of SSF (pre-incubated at a temperature of 37 °C), 5 μL of 0.3 M calcium chloride, 0.1 mL of amylase solution (1500 U·mL^−1^ in SSF) and 0.195 mL of distilled water were added, and vortexed. Subsequently, samples were placed in an overhead shaker (Reax 2, Heidolph, Germany), fitted inside a pre-heated drying cabinet (UNE300, Memmert, Germany) for 2 min at a temperature of 37 °C. Gastric phase was carried out with the addition of 1.28 mL of SGF (pre-incubated at a temperature of 37 °C), 1 μL of 0.3 M calcium chloride and 0.32 mL of pepsin solution (25,000 U·mL^−1^ in SGF). Between 58–63 μL of 6 M hydrochloric acid was added to the samples for hydrolysis and 0.336–0.341 mL of water was added accordingly to reach the end volume 4 mL. The mixture was incubated in the overhead shaker at a temperature of 37 °C for 2 h. For the small intestinal phase, 1.7 mL of SIF (pre-incubated at a temperature of 37 °C), 8 μL of 0.3 M calcium chloride, 0.5 mL of bile extract solution (160 mM in SIF, temperature 37 °C), 1.0 mL of pancreatin solution (800 U·mL^−1^ in SIF), 10 μL of 1 M sodium hydroxide and 0.782 mL of water were added to all samples. Mixtures were incubated in the overhead shaker at a temperature of 37 °C for another 2 h. Weight of digests was measured after completion of the small intestinal phase. After digestion, remaining intact proteins in the small intestinal digesta were precipitated with the addition of 32 mL of methanol and incubated at a temperature of −20 °C for 1 h. Pellet (undigested protein) was separated by centrifugation with 6000 rpm for 20 min at a temperature of 4 °C. Bioaccessible protein content was determined in acid-hydrolyzed supernatant. For this purpose, 50 μL of supernatant was transferred into chromatographic vials, evaporated by nitrogen gas and redissolved in hydrolysis buffer solution (260 μL of water, 120 μL of 0.1% dithiodipropionic acid/ 0.2 M sodium hydroxide, 120 μL of 0.2 M hydrochloric acid, 500 μL of 37% hydrochloric acid). Before closing, vials were flushed with nitrogen for 5 s, vortexed and incubated at a temperature of 110 °C for 15 h [88]. In vitro protein digestibility was assessed according to the OPA method [89], with minor modifications. Briefly, 580 μL of OPA reagent and 20 μL of acid-treated sample were mixed and kept in dark for 10 min. Absorbance was measured at 335 nm with a UV-spectrophotometer (Thermo Scientific^TM^, Waltham, MA, USA). Quantitative evaluation of amino acid was measure considering L-serine (0–125 mg·mL^−1^) as reference. Absorbance of samples were corrected with average absorbance value of the blank sample (protein-free biscuit). Protein digestibility was calculated by original protein content in soybean milk (30 g·L^−1^), measured by the Kjeldahl method [74] and bioaccessible protein content, measured by the OPA method.

$$\mathrm{In}\;\mathrm{vitro}\;\mathrm{protein}\;\mathrm{digestibility}\;\left(\%\right)=\frac{\mathrm{Bioaccesible}\;\mathrm{protein}\;\mathrm{content}}{\mathrm{Protein}\;\mathrm{content}\;\mathrm{in}\;\mathrm{soymilk}}\cdot 100$$

### 2.5. Statistical Analysis

All experiments were performed five times. The average value and standard deviation (S.D.) of experimental results was calculated by a Microsoft Excel spreadsheet (Microsoft Corporation, Washington, WA, USA). One-way analysis of variance (ANOVA) method, followed by the Tukey’s post hoc test were performed by SPSS 15.0 statistics software (IBM, Armonk, NY, USA) to determine the significant differences between different groups. The differences between groups were considered significant when *p* < 0.05.

## 3. Results and Discussion

### 3.1. Hydrolysis of SMP by Papain

**Figure 1:**
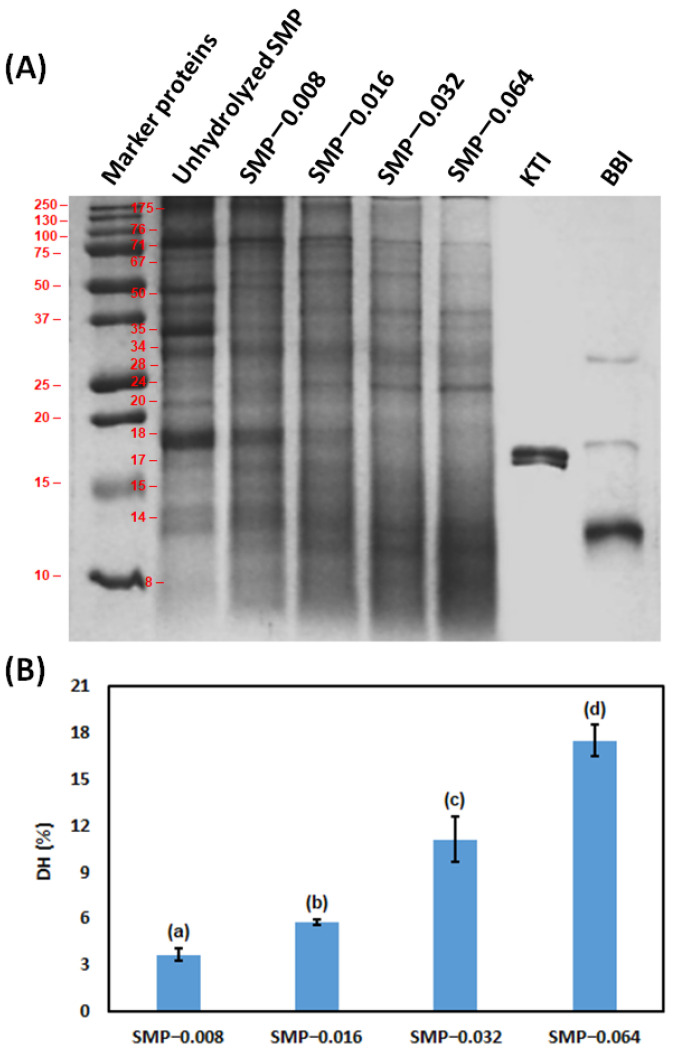
Image of SDS-PAGE of unhydrolyzed SMP papain-hydrolyzed SMP (**A**); DH of SMP using different concentrations of papain (**B**). The dissimilar alphabet in superscript represents the significant difference between results, evaluated by the Tukey’s post hoc method.

Prior to the papain hydrolysis of SMP, the concentrations of protein, carbohydrate, fat and pH in soybean milk were measured. Those values at RT were 30 ± 0.26 g·L^−1^, 26 ± 0.15 g·L^−1^, 17 ± 0.23 g·L^−1^ and 6.8, respectively. After inactivation of the papain, the total concentration of protein was not changed in a significant way, S.D. 1 × 10^−4^. In Figure 1A, proteins are presented in unhydrolyzed SMP and after their hydrolysis by papain in the sodium dodecyl sulfate–polyacrylamide gel. The original image of SDS-PAGE is represented in the Supplementary Section (Figure S1). In soybean milk, the two major protease inhibitors are KTI and BBI. However, KTI contributes ~74% and BBI contributes ~26% to the total trypsin inhibitory activity of raw soybean milk, heat treatment during soybean milk processing modulates their contribution [90]. Furthermore, KTI is also recognized as an IgE-binding protein [91]. Therefore, KTI and BBI were considered in lane seven and lane eight, respectively, in the sodium dodecyl sulfate–polyacrylamide gel.

Different proteins present in unhydrolyzed SMP are visualized in column two. The main types of protein in soybean are glycinin (11S) and β-conglycinin (7S), those contribute approximately 80% of the total protein in soybean. β-conglycinin is a trimer glycoprotein consisting of three glycosylated subunits, α′ (~71 kDa), α (~67 kDa) and β (~50 kDa) [92]. The hexameric glycinin is composed of five subunits with acidic and basic polypeptides, having molecular weight ~35 kDa and ~20 kDa, respectively [93,94]. Their presence in column two is noted. Some other proteins might inlcude lectin (~175 kDa) with SDS derivative peptide (~44 kDa) [95], Gly m 7 (~76 kDa) [96], P34 (~34 kDa) [97], P28 (~28 kDa) [98], KTI (2S) (~18 kDa) [99], Gly m 4 (~17 kDa) [100], Gly m 3 (~14 kDa) [101] and BBI (~8 kDa) [102]. Some researchers mentioned that BBI may exist with several isoforms. They can be produced from different genes [103], and also have the tendency towards self-association, which contributes to the formation of several forms of multimers (dimer-trimer-tetramer-hexamer) [103,104]. In column eight, it is noted that BBI conjugate has molecular weight of ~13 kDa, ~17 kDa and ~30 kDa. Furthermore, oleosin may present with different isoforms with molecular weight ~24 kDa and ~15 kDa [105]. Molecular weight of proteins in soybean milk can be altered due to heat-induced protein aggregation during soybean milk processing [106]. Papain hydrolysis of SMP samples, such as SMP-0.008, SMP-0.016, SMP-0.032 and SMP-0.064 are visualized in column three, column four, column five and column six, respectively. Hydrolysis of proteins was increased with increase in the concentration of papain in the hydrolysis reaction. The band intensities of different proteins are presented in Table S1, provided in the Supplementary Section. A peptide with molecular weight ~55 kDa was produced due to the hydrolysis of the mentioned proteins. Without any contradiction it is noted that peptides with low molecular weight were produced with an increase in the concentration of papain in the hydrolysis reaction. Therefore, the hydrolysis of proteins with lower molecular weight 14 kDa and 8 kDa are not easily understandable based on band intensity. In the present research, the concentration range of papain was 0.008–0.064 g·L^−1^. In this concentration range of papain, there was no protein precipitation. Protein coagulation and precipitation were observed when the concentration of papain was 0.096 g·L^−1^. Oil bodies in soybean milk are present in a stable manner because they are encapsulated by soybean protein oleosin. Intensive papain digestion of oil body-stabilizing oleosin endorsed oil body agglomeration with proteins and they were subsequently precipitated [107,108]. We found a similar result in our previous investigation [63].

In Figure 1B, DH of SMPs with different concentrations of papain is presented. DH of SMP was increased with an increase in the concentration of papain in the hydrolysis reaction in a significant way (*p* < 0.05). As an example, DH for SMP-0.008 was 3.6% (S.D. ± 0.4) and it became 17.5% (S.D. ± 1.0) for SMP-0.064. A higher concentration of papain was responsible for the hydrolysis of peptide bonds in native protein and release peptides. Several investigators reported hydrolysis of proteins in soybean protein isolate (SPI) by papain at different ratios of enzyme and substrate, temperature and time of hydrolysis [64,65,66,67,68,69,70]. The value of DH of protein depends on several factors, such as type of enzyme, activity of the enzyme, presence of a peptide bond in the protein, hydrolysis temperature, pH and time [109]. Furthermore, the value of DH depends on the analytical method. Therefore, results are not directly comparable in all situations [75,110]. 

### 3.2. Antioxidant Capacity

**Figure 2:**
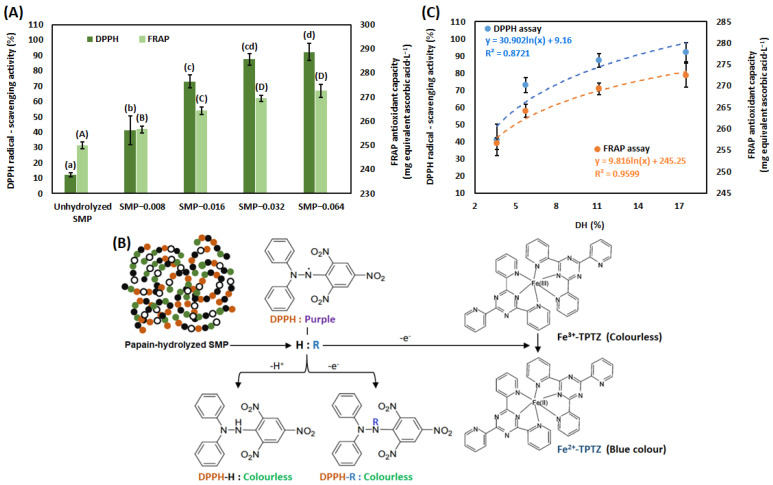
Antioxidant capacity of unhydrolyzed and papain-hydrolyzed SMP (**A**). Mechanisms of antioxidant capacity for papain-hydrolyzed SMP (**B**). Antioxidant capacity of papain-hydrolyzed SMP with respect of DH (%) (**C**). Results are presented by mean value with standard deviation (± values). The dissimilar alphabet in superscript represents the significant difference between results, evaluated by the Tukey’s post hoc method.

In Figure 2, the antioxidant capacity of unhydrolyzed SMP, SMP-0.008, SMP-0.016, SMP-0.032 and SMP-0.064 is shown. The antioxidant capacity of unhydrolyzed SMP and papain hydrolysis of SMP was measured by DPPH and FRAP assays. According to the DPPH assay, radical scavenging activity was 12.27% (S.D. ± 1.16) for unhydrolyzed SMP. It is noted that antioxidant capacity, measured by the DPPH radical scavenging assay was increased in a significant way due to the use of papain 0.008–0.064 g·L^−1^. According to the FRAP assay, antioxidant capacity was 249.96 mg, equivalent to ascorbic acid·L^−1^ (S.D. ± 1.34) for unhydrolyzed SMP. Antioxidant capacity, measured by FRAP assay, was increased in a significant way due to the use of papain 0.008–0.032 g·L^−1^.

Proteolytic activity of papain has specificity on valine, phenylalanine and tyrosine at P2 position, except Val in P1′ position [111]. The presence of hydrophobic amino acids (electron donor) in the peptide chain exhibits reducing activity toward Fe^3+^. Similarly, peptides with hydrophobic amino acids can convert DPPH free radical to a more stable molecule (radical scavenging activity) (Figure 2B). It was reported that smaller peptides can react with free radicals due to lower steric hindrance. Peptides with low-molecular weight have higher charge-to-mass ratio due to the presence of more electron-rich side chains than larger peptides. Therefore, lower molecular weight of peptides offers better reducing activity [112,113]. It was reported that peptides with cationic amino acid lysine (K) or arginine (R) at the C-terminal position contribute the radical scavenging activity [114]. Several peptides with K or R in C-terminal position, produced by papain hydrolysis of SPI were reported by Margatan with co-authors. Those are IPSQVQELAFPGSAQAVEKLLK, TEAQQSYLQGFSR, INAENNQRNFLAGEKDNVVR, ALPEEVIQHTFNLK, ATSLDFPALWLLK, IVRNLQGENEEEDSGAIVTVK, LDFPALWLLK, TSLDFPALWLLK and VSIIDTNSLENQLDQMPR [70]. It may feel that these peptides may offer antioxidant capacity in papain-hydrolyzed SMP. Antioxidant capacity, measured by the FRAP and DPPH assays is plotted with the DH of SMP (Figure 2C). It is noted that antioxidant capacity and DH of SMP is directly correlated. Correlations along with their strengths are mentioned in Figure 2C. Due to the hydrolysis of peptide bonds in SMP, electron-rich peptides were produced. They offered reducing activity toward Fe^3+^ and DPPH radical scavenging activity.

### 3.3. ACE-Inhibitory Activity

**Figure 3:**
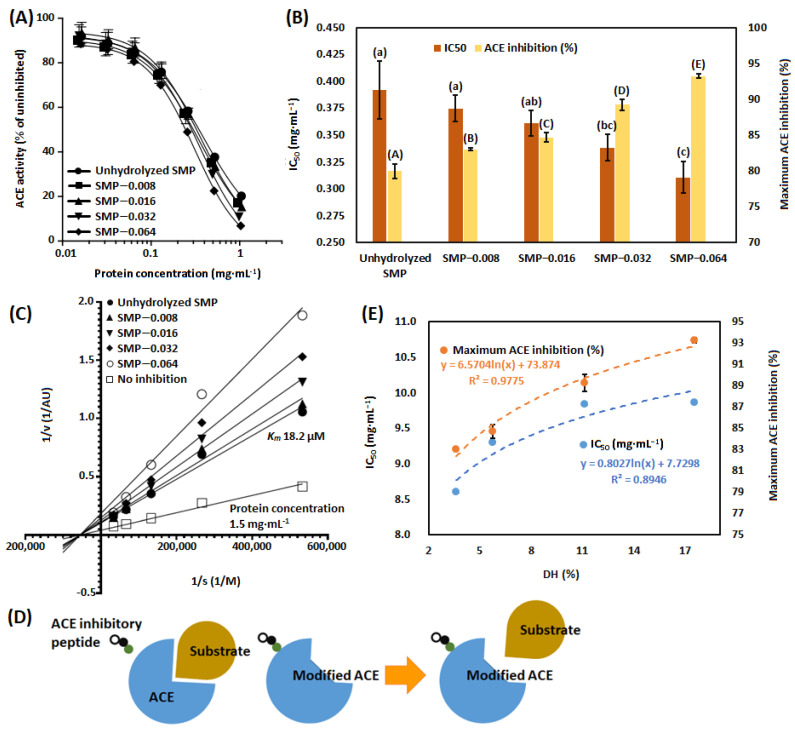
Uninhibited ACE activity (%) of unhydrolyzed and papain-hydrolyzed SMP (**A**). IC_50_ and maximum ACE inhibition (%) of unhydrolyzed and papain-hydrolyzed SMP (**B**). The Lineweaver–Burk plot of unhydrolyzed and papain-hydrolyzed SMP (**C**). The mechanism of ACE inhibition by peptide (**D**). Anti-angiotensin activity of papain-hydrolyzed SMP with respect of DH (%) (**E**). Results are presented by mean value with standard deviation (± values). The dissimilar alphabet in superscript represents the significant difference between results, evaluated by the Tukey’s post hoc method.

ACE inhibitory activities of unhydrolyzed SMP and papain-hydrolyzed SMP (SMP-0.008, SMP-0.016, SMP-032 and SMP-0.064) are presented in Figure 3A. From the figure it is noted that 100% inhibition of ACE is not achieved. This can be justified by the fact that peptides with anti-angiotensin activity, produced by papain hydrolysis of SMP might change the structural configuration of ACE, which might not be suitable for interaction between ACE and substrate [115]. Maximum inhibition and IC_50_ values of unhydrolyzed SMP and papain-hydrolyzed SMP are presented in Figure 3B. From the figure, it is noted that inhibition of ACE is dependent on the concentration of papain in the hydrolysis reaction. The application of papain in the proteolysis of SMP generates low-molecular weight of peptides and the binding sites for ACE are exposed. Low molecular weight of peptides has more chance to interact with ACE, due to less steric hindrance [116]. Pripp with co-authors reported that there was a positive correlation between the hydrophobicity of the C-terminal amino acid in peptide and anti-ACE activity [117]. Peptides with hydrophobic amino acids, such as phenylalanine (F), proline (P), tryptophan (W) and tyrosine (Y) at the C-terminal position can bind with ACE and offer anti-angiotensin activity [118]. Dellafiora with co-authors reported on tetra-peptide LPYP from SPI with anti-angiotensin activity [119]. Furthermore, it was reported that the presence of positively-charged amino acid lysine (K) or arginine (R) at the C-terminal position in peptide can also contribute to anti-angiotensin activity [114,120]. Margatan with co-authors reported that IC_50_ value of papain hydrolysis of SPI was 0.18 mg protein·mL^−1^, when 5% of substrate was hydrolyzed by 0.2 units·mL^−1^ of papain for 45 min at a temperature of 70 °C and pH 7. They identified several peptides with K or R in C-terminal position, produced by papain hydrolysis of SPI, supposed to have anti-angiotensin activity [70]. Lee with co-authors reported that inhibition of ACE was greater when SPI was hydrolyzed by papain than bromelain, flavozyme and neutrase. Inhibitions of ACE were 45%, 48%, 62% and 75% for papain-hydrolyzed SPI with DH 6%, 9%, 12% and 15%, respectively [68].

In Figure 3C, the Lineweaver–Burk plot was developed with enzymatic reaction velocity and corresponding substrate concentration. The graph demonstrates a non-competitive mechanism of ACE inhibition by papain hydrolysate of SMP. In the experiment, protein concentration 1.5 mg·mL^−1^ was used and the *K_m_* value was determined to be 18.2 µM. Peptides with low-molecular weight and amino acids with hydrophobic (F, P, W and Y) or positive charge (K and R) at the C-terminal position can bind with ACE and may change the conformation of the active site of the ACE. It reduces the interaction of ACE with substrate [70,118]. Inhibition mechanism of ACE by SMP-derived peptide is presented in Figure 3D. Vahid and Rotimi reported that cationic peptides, produced by the pepsin hydrolysis of SPI showed the inhibition of ACE by noncompetitive manner [114]. Anti-angiotensin activity, represented by values of IC_50_ and maximum inhibition of ACE are plotted with the DH of SMP (Figure 3E). Correlations along with their strengths are also mentioned. It is noted that anti-angiotensin activity and DH of SMP has positive correlation.

### 3.4. Antigenic Activity

**Figure 4:**
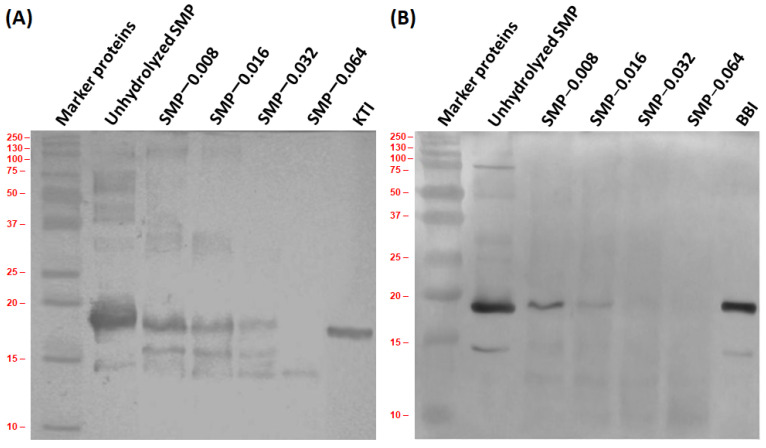
Immunoblot of unhydrolyzed and papain-hydrolyzed SMP with Rb polyclonal antibodies: KTI (**A**) and BBI (**B**).

SMP allergy is a complex disorder because most of the proteins in soybean contain a wide range of allergenic epitopes, including linear and conformational epitopes [35]. One of the major allergenic proteins in soybean is KTI, which contains IgE-binding epitopes and has two disulfide bridges. Another soy protein BBI has seven disulfide bridges [121]. Antigenicity of unhydrolyzed SMP, SMP-0.008, SMP-0.016, SMP-0.032 and SMP-0.064 were studied using Rb polyclonal antibodies against KTI (Figure 4A) and BBI (Figure 4B). Original images of immunoblots for KTI and BBI are represented in the Supplementary Section (Figure S2).

The immunoblot for KTI is presented in Figure 4A. Compared to column seven, it may say that KTI in SMP has interaction with the antibody Rb anti-KTI (column two of Figure 4A). Furthermore, some cross-reactivity with other proteins is shown in column two. They might be lectin (~175 kDa) with its SDS derivative peptide (~44 kDa), α′ (~71 kDa), α (~67 kDa) and β (~50 kDa) subunits of β-conglycinin, acidic polypeptide of glycinin (~35 kDa) and Gly m 3 (~14 kDa). It is noted that antigenicity of KTI is decreased with increase in the concentration of papain. Cross-reactivity with other proteins with molecular weights of ~175 kDa, ~44 kDa, ~35 kDa and ~14 kDa are also shown in SMP-0.008. A new antigenic peptide with molecular weight ~16 kDa is produced when SMP was treated with 0.008 g·L^−1^ of papain (column three). Its hydrolysis is increased with the increase in the concentration of papain. Antigenic activities of proteins with molecular weights of ~175 kDa and ~35 kDa are almost reduced at SMP-0.032 (column five). Reduction in the antigenicity of other proteins and peptides are almost reduced in SMP-0.064, except the protein with molecular weight ~14 kDa (column six). 

The immunoblot for BBI is presented in Figure 4B. However, a BBI conjugate with molecular weight ~30 kDa is noted in column eight of SDS-PAGE (Figure 1A), its interaction with the antibody Rb anti-BBI is not seen in column seven of immunoblot. Compared to column seven, it may be seen that two different BBI conjugates with molecular weight ~17 kDa and ~14 kDa have interactions with the antibody Rb anti-BBI. In column two, cross-reactivity with other proteins are also noted. Those proteins might be Gly m 7 (~76 kDa), β-subunits of β-conglycinin (~50 kDa), P28 (~28 kDa) and oleosin (~24 kDa). They are almost reduced in SMP-0.008. Due to hydrolysis of SMP, a new peptide (~12) having cross-reactivity with the antibody Rb anti-BBI is produced (column three). Its hydrolysis is dependent on the concentration of papain. Antigenic activities of BBI conjugates with molecular weight ~17 kDa and ~14 kDa are reduced with increase in the concentration of papain in the hydrolysis reaction. Antigenic activities of them were almost reduced in SMP-0.064. 

### 3.5. Digestibility

**Figure 5:**
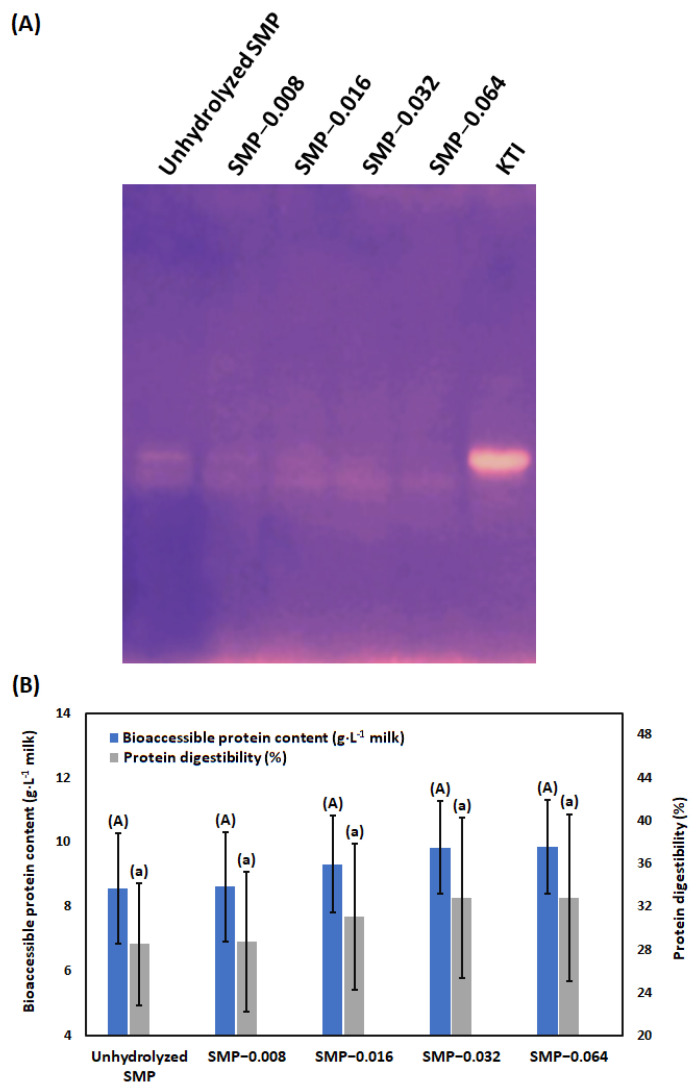
Inhibitory activity of KTI in unhydrolyzed and papain-hydrolyzed SMP in native (non-denaturing condition) polyacrylamide gel (**A**), bioaccessible protein content and protein digestibility of unhydrolyzed and papain-hydrolyzed SMP in vitro digestion (**B**). Results are presented by mean value with standard deviation (± values). The dissimilar al-phabet in superscript represents the significant difference between results, evaluated by the Tukey’s post hoc method.

Prior to in vitro digestibility study, activities of KTI and BBI in unhydrolyzed and papain-hydrolyzed SMP were studied in native PAGE. Both KTI and BBI belong to the serine proteinase inhibitor family, accounting for 1.4% and 0.6% of soybean protein, respectively [122]. KTI is the primary inhibitor of trypsin; whereas BBI is an inhibitor for both trypsin and chymotrypsin [8]. In our investigation, interaction between chymotrypsin with unhydrolyzed and papain-hydrolyzed SMP in native gel was not found. Contradictorily, interaction between trypsin with unhydrolyzed and papain-hydrolyzed SMP in native gel was noted (Figure 5A). The original image of native polyacrylamide gel for inhibitory activity of KTI is represented in the Supplementary Section (Figure S3). The interaction between trypsin and SMP was reduced with an increase in the concentration of papain in the hydrolysis reaction. However, there was a common belief that BBI is more stable than KTI due to the presence of multiple intrachain disulfide bonds [90], some pioneering researchers reported that KTI can be more thermostable than BBI [102]. Other investigators reported that protease inhibitory effect of BBI in soy flour was absent; whereas the activity of KTI was present in soy flour [123]. According to our result, it may be realized that while BBI was present in unhydrolyzed SMP, its binding site with chymotrypsin was destroyed by heat treatment during processing. 

According to Figure 5A, hydrolysis of KTI in SMP was increased due to treatment with papain in a concentration-dependent manner. Concentration of bioaccessible amino acid and protein digestibility in unhydrolyzed and papain-treated soybean milk are presented in Figure 5B. It is noted that with increase in the concentration of papain for hydrolysis of SMP, bioaccessible amino acid content and in vitro protein digestibility are not statistically increased. It was reported that KTI can be inactivated by acidic gastric juice [124]. Hydrolysis of SMP by papain produces peptides with low molecular weight, which are further hydrolyzed by pepsin and gastric hydrochloric acid in gastric phase. Subsequently, they are converted to amino acids by the actions of trypsin and chymotrypsin, present in pancreatic juice. 

## 4. Conclusions

Consumption of soybean milk has some limitations because several proteins with linear and conformational epitopes in protein structure may cause food anaphylaxis in the SMP-sensitive community. KTI is a labile protein in the gastrointestinal tract and has an immunogenic activity. Furthermore, BBI can inhibit the activity of both trypsin and chymotrypsin in the gastrointestinal tract. Therefore, high levels of protease inhibitors in soybean-based products could decrease protein digestibility and might cause pancreatic disease. Presently, allergen-free food products with functional activity have grabbed lots of attention. In the present investigation, effects of the treatment of papain on hydrolysis of SMP were investigated. Alternation of IgE-binding epitopes and functional activities (antioxidant and anti-ACE) after papain hydrolysis of SMP were taken into consideration. 

SMP was hydrolyzed by papain with different concentrations, such as 0.008 g·L^−1^ (SMP-0.008), 0.016 g·L^−1^ (SMP-0.016), 0.032 g·L^−1^ (SMP-0.032) and 0.064 g·L^−1^ (SMP-0.064). According to the SDS-PAGE, peptides with lower molecular weight were produced by increasing the hydrolysis of SMP by papain. Without any contradiction, DH was increased in a significant way with increase in the concentration of papain in the hydrolysis reaction. Peptides with K or R in C-terminal position were produced by papain hydrolysis of SMP. They offered antioxidant capacity, measured by the ferric-reducing ability of plasma (FRAP) and 2,2-Diphenyl-1-picrylhydrazyl (DPPH) assays. Presence of hydrophobic amino acids in C-terminal position of peptides offered anti-angiotensin activity, measured by recombinant angiotensin converting enzyme and substrate Abz-FRK(Dnp)-P. Mechanism of the anti-angiotensin activity by SMP hydrolysate was non-competitive. Direct correlations were observed between antioxidant capacity and anti-angiotensin activity with DH of SMP. Antigenicity of KTI and BBI was measured by the Rb polyclonal antibodies against KTI and BBI together with peroxidase-labelled goat anti-Rb IgG secondary antibody. Antigenicity of both KTI and BBI was reduced in SMP by increasing the concentration of papain. However, there was a common belief that BBI is more stable than KTI, our experimental results present that the binding potentiality of BBI with chymotrypsin was destroyed. This might be due to heat treatment during processing. The binding potentiality of protease inhibitors with trypsin was reduced with an increase in the hydrolysis of SMP by papain. In vitro digestion simulation of unhydrolyzed SMP and papain-hydrolyzed SMP was carried out according to the Infogest consensus method. According to the result, in vitro protein digestibility was not statistically increased with an increase in the concentration of papain. 

The present research was performed in a laboratory-scale setup. In this research, the antigenicity of KTI and BBI in unhydrolyzed and papain-hydrolyzed SMP was measured by Rb polyclonal anti-KTI and anti-BBI antibodies together with peroxidase-labelled goat anti-Rb IgG secondary antibody. It was noted that antigenicity of some proteins or peptides were still present at SMP-0.064. To confer the proper concentration of papain and propose the concept of producing allergen-free soybean milk for human consumption, further investigation related with allergenic activity is needed with SMP-positive pooled human serum. In the present investigation, we tried to explain the antioxidant capacity and anti-angiotensin activity of peptides by already-published amino acid sequences. It may seem that further investigation is needed for the comprehensive identification of peptides with antioxidant capacity and anti-angiotensin activity from SMP. In further investigations, bioinformatics tools will be taken into consideration. Furthermore, other food-grade proteolytic enzymes, such as bromelain, ficin, zingibain, actinidin and cardosin for hydrolysis of SMP, prior to in vitro digestion, can be assessed in future research.

We believe that the results from the laboratory-scale setup will provide an initial idea of the production of papain-hydrolyzed SMP in an industrial scale. It is supposed that the present research may reduce the limitation of consumption of soybean milk and soybean-based products. Furthermore, it is expected that the findings of the present research may receive attention from both the academic sector and the food industry.

## Data Availability

Not applicable.

## Supplementary Materials

The following supporting information can be downloaded at: https://www.mdpi.com/article/10.3390/bioengineering9090418/s1, Figure S1: Image of SDS-PAGE of unhydrolyzed SMP papain-hydrolyzed SMP; Figure S2: Immunoblot of unhydrolyzed and papain-hydrolyzed SMP with Rb polyclonal antibodies: KTI (A) and BBI (B); Figure S3: Inhibitory activity of KTI in unhydrolyzed and papain-hydrolyzed SMP in native (non-denaturing condition) polyacrylamide gel; Table S1: Band intensities of different proteins in unhydrolyzed SMP papain-hydrolyzed SMP in the sodium dodecyl sulfate–polyacrylamide gel.
