# Supplementary material for: Hydrolysis of Soybean Milk Protein by Papain: Antioxidant, Anti-Angiotensin, Antigenic and Digestibility Perspectives
Source: Bioengineering (Basel). 2022 Aug 26;9(9):418. doi: 10.3390/bioengineering9090418 (PMC9495856; doi:10.3390/bioengineering9090418)
Supplement: Supplementary file 1 [file bioengineering-09-00418-s001.zip › bioengineering-1855455-SI.pdf]

Figure S1

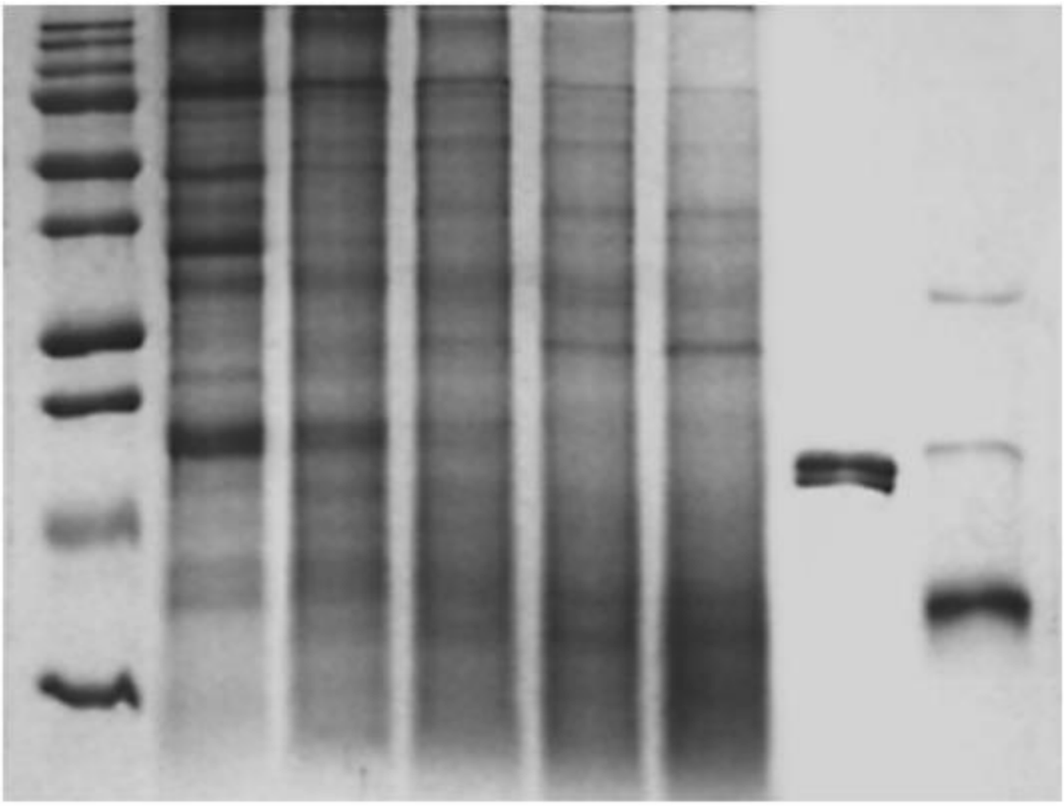

Figure S2

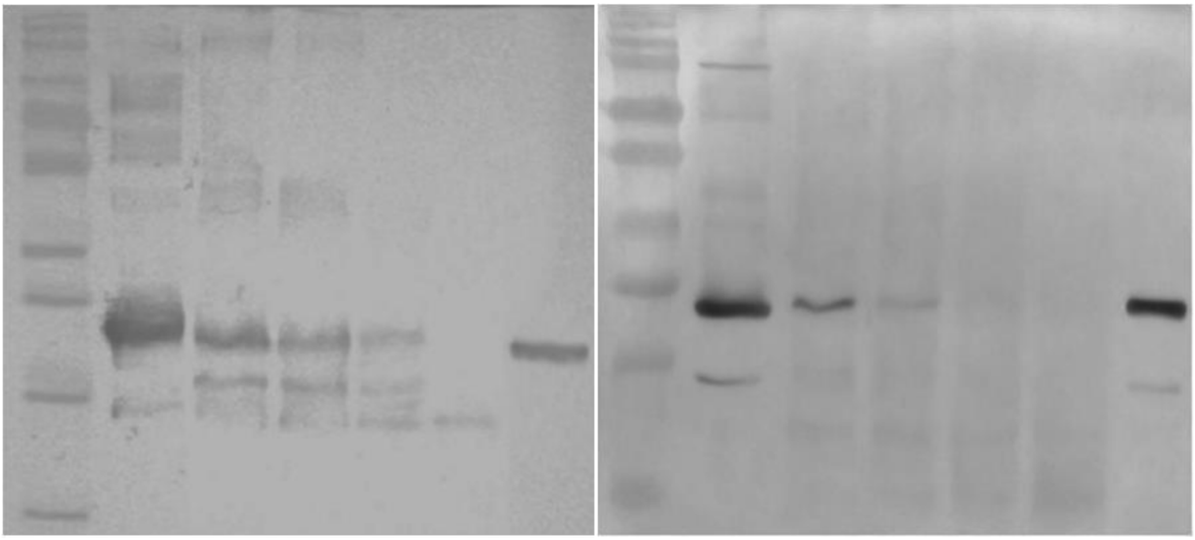

Figure S3

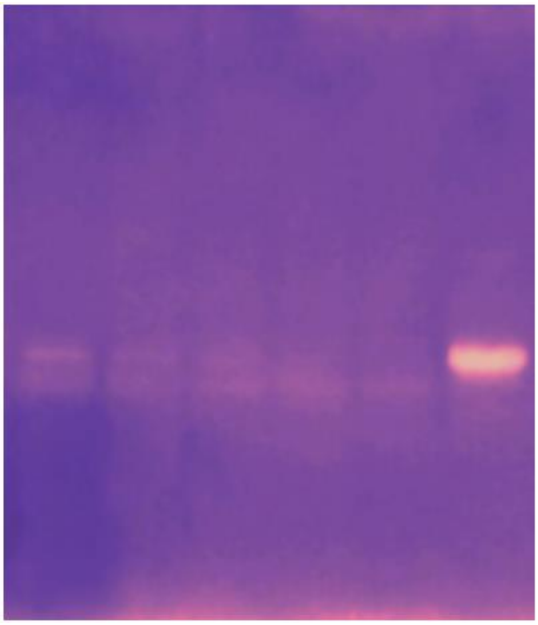

Table S1

	175 kDa	76 kDa	71 kDa	67 kDa	50 kDa	44 kDa	35 kDa	34 kDa	28 kDa	24 kDa	20 kDa
Unhydrolyzed SMP	23011.761	21173.267	21679.033	20319.196	24373.317	21957.167	24787.468	22846.803	20465.773	18948.702	21784.652
SMP-0.008	21565.832	20288.782	20422.146	19086.196	19256.589	18709.711	16296.125	20237.418	20239.539	23171.267	20928.752
SMP-0.016	15688.246	15929.317	17287.246	16844.004	15958.832	17684.246	14317.882	18060.711	20160.539	22294.489	17211.004
SMP-0.032	8624.882	10163.196	12569.075	12647.196	11700.317	16062.903	12468.024	15052.146	18939.924	22438.146	15780.782
SMP-0.064	2355.033	3026.640	5323.832	6048.397	6679.903	11754.004	11107.832	13864.974	17916.924	23621.338	15401.388

Table S1 Continue

	18 kDa	14 kDa	8 kDa
Unhydrolyzed SMP	27892.075	16034.146	6734.660
SMP-0.008	22240.196	21817.773	12917.631
SMP-0.016	17624.660	22230.317	17136.024
SMP-0.032	14619.075	21577.489	17847.095
SMP-0.064	14312.731	25761.844	27016.572
